# Modeling Count Outcomes from HIV Risk Reduction Interventions: A Comparison of Competing Statistical Models for Count Responses

**DOI:** 10.1155/2012/593569

**Published:** 2012-03-25

**Authors:** Yinglin Xia, Dianne Morrison-Beedy, Jingming Ma, Changyong Feng, Wendi Cross, Xin Tu

**Affiliations:** ^1^Department of Biostatistics and Computational Biology, Box 630, University of Rochester, 265 Crittenden Boulevard, Rochester, NY 14642, USA; ^2^College of Nursing, University of South Florida, 12901 Bruce B. Downs Boulevard, MDC22, Tampa, FL 33612, USA; ^3^Department of Psychiatry, University of Rochester, 300 Crittenden Boulevard, Rochester, NY 14642, USA

## Abstract

Modeling count data from sexual behavioral outcomes involves many challenges, especially when the data exhibit a preponderance of zeros and overdispersion. In particular, the popular Poisson log-linear model is not appropriate for modeling such outcomes. Although alternatives exist for addressing both issues, they are not widely and effectively used in sex health research, especially in HIV prevention intervention and related studies. In this paper, we discuss how to analyze count outcomes distributed with excess of zeros and overdispersion and introduce appropriate model-fit indices for comparing the performance of competing models, using data from a real study on HIV prevention intervention. The in-depth look at these common issues arising from studies involving behavioral outcomes will promote sound statistical analyses and facilitate research in this and other related areas.

## 1. Introduction

Analysis of sexual behavioral outcomes, especially count data, can be challenging even for experienced investigators [1]. Count data from sexual behaviors are often characterized with many zeros and overdispersion, creating quite complex challenging issues for modeling such data. For example, we recently conducted a randomized control study to test the efficacy of a prevention intervention for reducing sexually transmitted infections (STIs)/HIV infections in African American adolescent girls living in an urban environment, a high-risk group bearing considerable health burdens from unprotected sex including increased risk for sexually transmitted infections (STIs) including HIV (PI: Dr. Morrison-Beedy). The primary outcomes, such as the number of unprotected vaginal sex experiences over the past 3 months, all had a preponderance of zeros and overdispersed variance, violating the assumptions of the Poisson distribution, the most popular statistical model for count responses. The primary hypothesis of the study is that the adolescent girls receiving the HIV risk reduction intervention in the study would reduce risky sexual behaviors as compared to the girls in the control condition. Although alternative statistical models are available for addressing both these methodological issues, the relative strengths and advantages of one model over its competitors and how to assess such model specific traits have not been thoroughly discussed in the extant literature.

Our objective in this article is three fold. First, we want to raise awareness of the two aforementioned statistical issues underlying the count outcome data arising from HIV prevention intervention studies that too often have either been completely ignored or dealt with using ad-hoc methods. Second, we focus on the etiology of the two key issues and compare four popular statistical models for addressing the underlying causes of the problems, laying the conceptual foundation for comparing these competing models when applied to real studies in HIV prevention intervention research. Finally, we illustrate the conceptual differences across the four models using data from a large NIH-funded study on testing the efficacy of a prevention intervention for reducing STI/HIV infections in a high-risk population.

We will (a) discuss choosing an appropriate model suitable for the analysis of many zero-valued and over-dispersed HIV risk reduction intervention data, (b) evaluate the four popular count data models using actual sexual behavior data, and (c) identify the need for further methodological and modeling approaches.

In Section 2, we briefly describe the design of the HIV risk reduction intervention study and the primary outcomes. In Section 3, we discuss four models (Poisson, NB, ZIP, and ZINB) for such count outcomes, major conceptual differences across them, and popular statistics for assessing goodness of fit statistics to help select appropriate and optimal models for the data at hand. In Section 4, we compare the performance of these models using the goodness of fit measures introduced along with plots of observed versus fitted values, and estimates of parameters from the Poisson, NB, ZIP, and ZINB models. In Section 5, we discuss the major implications of the results as well as issues for further methodological research.

## 2. Data and Study Design

 The data used to compare and demonstrate differences across the four models for count outcomes, Poisson, NB, ZIP and ZINB, comes from the incidental sexual behavior of adolescent girls collected in the HIV risk reduction intervention funded by the study (NR R01008194, PI: Dr. Morrison-Beedy). We start with a brief description of the study population and its major outcomes.

### 2.1. Study Participants

 The study participants were 639 sexually active girls 15–19 years of age. Eligibility criteria included: (a) unmarried, (b) not pregnant, (c) had not delivered a child within the past 3 months, (d) reported sexual intercourse (vaginal, anal, or oral) with a male in the past three months, and (e) able to participate in an English-speaking intervention. Girls were recruited from adolescent health clinics, youth development centers, and school-based health centers located in upstate New York as well as self-referred through word-of-mouth.

 Of the 1778 girls who were screened, over 765 (43%) did not meet the eligibility criteria. From the 1013 that were eligible, 738 consented. In some cases, initial protracted waiting times to their first intervention session resulted in fewer consenters actually attending groups. Of those who attended, 329 (51%) were randomized to the HIV intervention group and 310 (49%) to the control group. Following IRB approvals from all participating institutions, we obtained a Federal Certificate of Confidentiality to further protect participants' privacy during the course of the study and also registered the trial at http://ClinicalTrial.gov/. A parental waiver of consent was granted by the IRBs, as supported by New York State law, which allows 14–17 year olds to seek reproductive health care without parent consent. Participants were enrolled from late December 2004 to April 2008, with intervention groups starting in January 2005.

### 2.2. Data Collection

 Study participation involved 6 group intervention sessions and 6 data collection points including a baseline assessment. Most participants began attending group intervention sessions within 4 weeks of enrollment. Assessments were scheduled for the study subjects from enrollment to 12 months followup. The battery of instruments were collected by 10–30 minute audio computer-assisted self-interview (ACASI) at baseline, and 1 week, 3 months, 6 months, and 12 months postintervention.

### 2.3. Sexual Risk Behavior and Distribution of Primary Outcomes

Items to assess sexual risk behavior were adapted from previous research [2–4] and included the number of male sexual partners (lifetime and past 3 months), and the number of episodes of protected and unprotected vaginal and anal sex (past 3 months) with steady and nonsteady (i.e., casual, infrequent, anonymous) partners. Consistent with prior recommendations [1], responses were summed to determine participants' number of episodes (a) of protected and (b) unprotected sex (vaginal and anal) in the past 3 months with steady and nonsteady partners.

To assess the effect of the intervention, the primary behavioral outcomes were reported incidents of (1) all vaginal sex episodes (regardless of partner type or condom use status), (2) unprotected vaginal sex with steady partners, (3) unprotected vaginal sex with other partners, and (4) any unprotected vaginal sex with steady or other partners, at each of the 3, 6, and 12 months followups.

**Figure 1:**
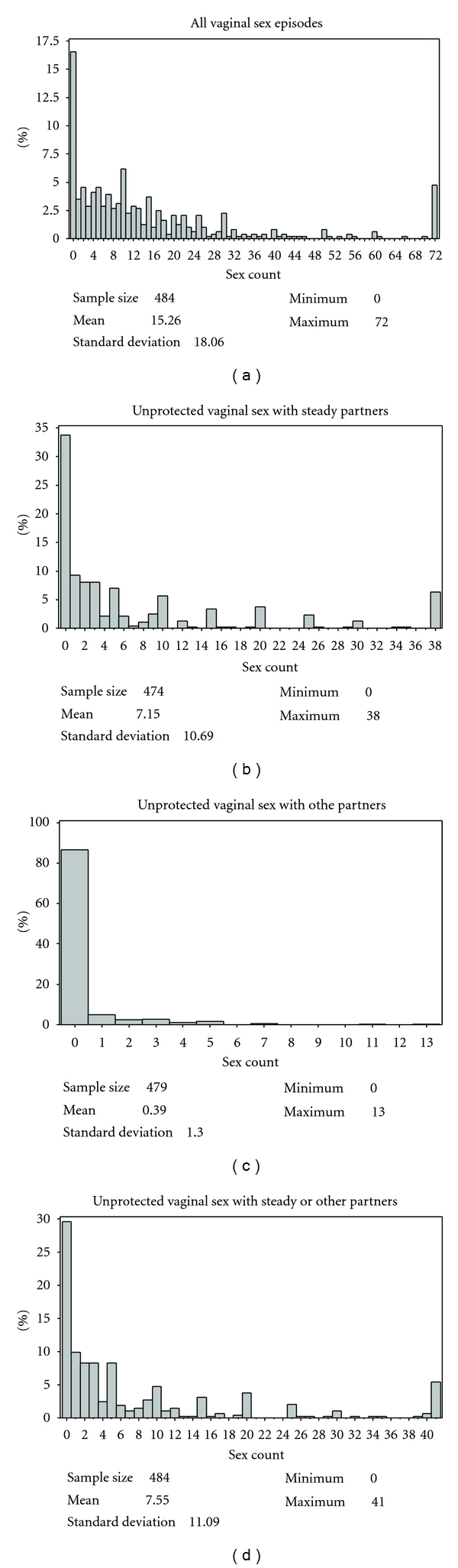
Distribution of outcome variables at 12 months.

 As shown in Figure 1, the HIV risk reduction intervention data are characterized by many zero-valued observations and a long right tail for outcomes at 12 months. The distribution patterns for 3- and 6-month outcomes are similar and not displayed. As shown in Table 1, the percentage with no reported sex in the past 3 months at 3, 6, and 12 months followup are 16.35%, 17.90%, and 16.28% for all vaginal sex episodes; are 43.67%, 42.38%, and 33.69% for unprotected vaginal sex with steady partners; are 87.69%, 89.61% and 86.50% for unprotected vaginal sex with other partners; and are 39.47%, 39.67%, and 29.44% for unprotected vaginal sex with steady or other partners. Overall, the percents have decreased sharply for all four outcomes from 3 to 12 months, especially for the unprotected vaginal sex with steady partners and unprotected vaginal sex with steady or other partners' outcomes. For each of the four outcomes, the variance is much larger than its mean (see Table 1), indicating overdispersion in the data.

## 3. Statistical Methods

### 3.1. Poisson, NB, ZIP, and ZINB Distributions

#### 3.1.1. Poisson Regression

 Poisson regression is the most popular regression model for count data. It assumes that each observed count *Y*_*i*_ is sampled from a Poisson distribution with the conditional mean *μ*_*i*_ given a vector of covariates/predictors *X*_*i*_ for each *i*th subject. The Poisson distribution, derived based on modeling the number of independent events from a memory-less Poisson process with a constant event rate, has the following density function:

(1) $$\begin{array}{c} P\left(Y_{i}=y\;|\;X_{i}\right)=\frac{\exp\left(-\mu_{i}\right){\;\mu }_{i}^{y}}{y!}, \end{array}$$

where *μ*_*i*_ = exp⁡(*X*_*i*_^*T*^ *β*).

A distinctive feature of the Poisson is the equality of the variance and mean,Var⁡(*Y*_*i*_ | *X*_*i*_) = *μ*_*i*_, which unfortunately also becomes a major limitation of this model in applications. For example, within our context, the multiple behavioral events from the same person over a period of time such as unprotected vaginal sex are highly correlated, resulting in a larger variance Var⁡(*Y*_*i*_ | *X*_*i*_) than its mean *μ*_*i*_ = exp⁡(*X*_*i*_^*T*^ *β*), a phenomenon known as overdispersion. When overdispersion is an issue, the estimates based on Poisson regression will be inefficient [5].

Some software packages such as SAS permit estimation of a dispersion parameter *α* to accommodate overdispersion. For example, both the SAS GENMOD and GLIMMIX procedures allow the modification of the Poisson model by including a dispersion parameter *α* to account for such overdispersion. With this technique, Var⁡(*μ*_*i*_) = *αμ*_*i*_  (where *α* > 0), when *α* < 1, the variance is less than its mean, indicating underdispersion, while for *α* > 1, the variance is larger than its mean, implying overdispersion in the data. This approach is ad hoc in the sense that it addresses overdispersed Poisson distribution at the “back end” estimation stage, rather than at the “front end” by explicitly modeling the overdispersion such as these we discuss next.

#### 3.1.2. Negative Binomial Regression

 As the most common alternative to Poisson regression, the negative binomial (NB) regression model addresses overdispersion by explicitly modeling the correlated events via a latent variable. Specifically, NB extends the Poisson by positing that the conditional mean *μ*_*i*_ of *Y*_*i*_ is not only determined by *X*_*i*_ but also by a heterogeneity (latent) component *e*_*i*_ independent of *X*_*i*_. If we assume that exp⁡(*e*_*i*_) is distributed with a gamma (1/*α*, 1/*α*), we obtain the NB model with the following density function:

(2) $$\begin{array}{c} P\left(Y_{i}\;|\;X_{i}\right)=\frac{\Gamma \left(Y_{i}+1/\alpha \right)}{\Gamma \left(Y_{i}+1\right)\Gamma \left(1/\alpha \right)}{\left(\frac{1/\alpha }{1/\alpha +\mu_{i}}\right)}^{1/\alpha }{\left(\frac{\mu_{i}}{1/\alpha +\mu_{i}}\right)}^{Y_{i}}, \end{array}$$

where *μ*_*i*_ = exp⁡(*X*_*i*_^*T*^ *β* + *e*_*i*_) = exp⁡(*X*_*i*_^*T*^ *β*)exp⁡(*e*_*i*_).

Since *E*(exp⁡(*e*_*i*_)) = 1, *E*(exp⁡(*X*_*i*_^*T*^ *β* + *e*_*i*_)) = *E*(exp⁡(*X*_*i*_^*T*^ *β*)), that is, whether we assume a Poisson or a negative binomial distribution, the expected value of *μ*_*i*_ does not change. However, since *α* > 0, under the negative binomial distribution, Var⁡(*Y*_*i*_ | *X*_*i*_) = *μ*_*i*_(1 + *αμ*_*i*_) > *μ*_*i*_. Therefore, Var⁡(*Y*_*i*_ | *X*_*i*_)/*E*(*Y*_*i*_ | *X*_*i*_) = 1 + *αμ*_*i*_. That is, the variance of the NB is greater than its mean, making provision for overdispersion.

Note that NB and Poisson models may be viewed as nested because as *α* approaches 0, NB approaches the Poisson.

#### 3.1.3. Zero-Inflated Poisson

 Although capable of addressing overdispersion, NB is not appropriate for modeling the data with a high percentage of zero counts as in the current context. To model such excess of zeros, zero-inflated Poisson regression may be applied [5, 7, 9, 10]. ZIP regression models originated in the econometrics literature [6], but their use has become more widespread, particularly since the publication of Lambert in 1992 [7]. ZIP is a mixture of two statistical processes, with one always generating zero counts and the other both zero and nonzero counts. That is, it assumes that each observation comes from one of two potential distributions, with one (group 1) consisting of a constant zero while the other (group 2) following Poisson. In a ZIP model, a logit model is typically used to model the probability of the constant zero, or structural zero, while the count data is modeled by the Poisson regression.

 Thus, two kinds of zeros are modeled by this mixture model: the sampling zeros due to sampling variability under Poisson and the structural zeros above and beyond the expected zero frequency under Poisson. In other words, an observed zero is generated by either the logistic process or the Poisson process.

Specifically, let *ω*_*i*_ = Pr⁡(*i* ∈ group  1  (structural  zero) | *Z*_*i*_) and 1 − *ω*_*i*_ = Pr⁡(*i* ∈ group  2  (sampling  zero) | *Z*_*i*_).

 Then, ZIP has the following distribution:

(3) $$\begin{array}{c} \begin{array}{c} P\left(Y_{i}\;|\;X_{i},Z_{i}\right)=\omega_{i}+\left(1-\omega_{i}\right)\exp\left(-\mu_{i}\right)\;\text{for}\;Y_{i}\;=\;0 \end{array},\\ \begin{array}{c} P\left(Y_{i}\;|\;X_{i},Z_{i}^{}\right)=\left(1-\omega_{i}\right){\frac{\exp\left(-\mu_{i}\right)\left(\mu_{i}\right)}{Y_{i}!}}^{Y_{i}}\;\text{for}\;Y_{i}>0 \end{array}, \end{array}$$

where *Z*_*i*_ and *X*_*i*_ are two sets of covariates linked to the logit and count data modules by Log(*ω*_*i*_/(1 − *ω*_*i*_  )) = *Z*_*i*_ *γ*, and Log(*μ*_*i*_) = *X*_*i*_ *β*. It is clear from (3) that the observed zeros come from the two sources of structural and sampling zeros.

 The mean and variance of *Y*_*i*_ are given by

(4) $$\begin{array}{c} \begin{array}{c} E\left(Y_{i}\;|\;X_{i},Z_{i}\right)=\omega_{i}0+\mu_{i}\left(1-\omega_{i}\right)=\mu_{i}\left(1-\omega_{i}\right), \end{array}\\ \begin{array}{c} \mathrm{Var}\left(Y_{i}\;|\;X_{i},Z_{i}\right)=\mu_{i}\left(1-\omega_{i}\right)\left(1+\mu_{i}\omega_{i}\right). \end{array} \end{array}$$

By (4), Var⁡(*Y*_*i*_ | *X*_*i*_, *Z*_*i*_)/*E*(*Y*_*i*_ | *X*_*i*_, *Z*_*i*_) = 1 + *μ*_*i*_ *ω*_*i*_ = 1 + [*ω*_*i*_/(1 − *ω*_*i*_)]*E*(*Y*_*i*_ | *X*_*i*_, *Z*_*i*_). Therefore, if *ω*_*i*_ approaches zero, that is, the amount of structural zeros decreases to zero, ZIP reduces to Poisson.

#### 3.1.4. Zero-Inflated Negative Binomial Regressions

By replacing the Poisson in ZIP with the negative binomial, we obtain the zero-inflated negative binomial, or ZINB. Thus, a ZINB has the general form:

(5) $$\begin{array}{c} \begin{array}{c} P\left(Y_{i}\;|\;X_{i},Z_{i}\right)=\omega_{i}+\left(1-\omega_{i}\right)g\left(\mu_{i}\right),\;\text{if}\;Y_{i}=\;0,\; \end{array}\\ P\left(Y_{i}\;|\;X_{i},Z_{i}\right)=\left(1-\omega_{i}\right)f\left(\mu_{i}\right),\;\text{if}\;Y_{i}>\;0, \end{array}\;\;$$

where *g*(*μ*_*i*_) = *P*(*Y*_*i*_ = 0 | *X*_*i*_) in the count data model, and  *f*(*μ*_*i*_) is the density of the negative binomial distribution. The binary process can be modeled using either logit or probit or other models for binary outcomes. The mean and variance of the ZINB are

(6) $$\begin{array}{c} E\left(Y_{i}\;|\;X_{i},Z_{i}\right)=\mu_{i}\left(1-\omega_{i}\right),\\ \mathrm{Var}\left(Y_{i}X_{i},Z_{i}\right)=\mu_{i}\left(1-\omega_{i}\right)\left(1+\mu_{i}\left(\omega_{i}+\alpha \right)\right) \end{array}$$

It follows from (6) that

(7) $$\begin{array}{cc} \frac{\mathrm{Var}\left(Y_{i}\;|\;X_{i},Z_{i}\right)}{E\left(Y_{i}\;|\;X_{i},Z_{i}\right)} & =1+\mu \left(\omega_{i}+\alpha_{i}\right)\\ & =1+\left[\frac{\omega_{i}+\alpha_{i}}{1-\omega_{i}}\right]E\left(Y_{i}\;|\;X_{i},Z_{i}\right). \end{array}$$

For ZINB, Var⁡(*Y*_*i*_ | *X*_*i*_, *Z*_*i*_) > *E*(*Y*_*i*_ | *X*_*i*_, *Z*_*i*_), demonstrating that ZINB also has the capability to model overdispersion. Since (*ω*_*i*_ + *α*_*i*_)/(1 − *ω*_*i*_) is a function of both zero-inflated parameter  *ω* and dispersion parameter  *α*, ZINB accounts for both population heterogeneity (mixture) and overdispersion in the distribution of the NB component of ZINB. Thus, NB is capable to model overdispersion due to unobserved heterogeneity; ZIP focuses on the violation of the Poisson by the population heterogeneity in the presence of structural zeros, while ZINB addresses both sources of heterogeneity.

 As shown in Figure 1 and Table 1, all the major study outcomes show a large percent of zeros, which along with the potential of overdispersion does not lend the analysis of these data to the traditional Poisson log-linear model. 

Within our context,  *ω* models the nonrisk subgroup of adolescent girls as represented by the structural zeros, while *μ* models the at-risk subgroup comprised of the positive response and sampling zeros. The nonrisk  *ω* is modeled the same way using a logistic model in both ZIP and ZINB, but the at-risk subgroup is modeled differently: by Poisson for ZIP, by NB for ZINB to account for overdispersion.

Besides empirical evidence, the appropriateness of ZIP and ZINB for modeling HIV risk reduction prevention data can also be argued on conceptual grounds. For example, within our context, all the adolescent girls were sexually active at baseline. However, it is plausible that some became abstinent, especially for those in the intervention group. Thus, at the followup visit, each subject belongs to one of the two groups, with one consisting of sexually active girls, and the other abstinent girls. The subjects in the first group had no sex during the given period although they were sexually active. Those in the second group also had no sex in the previous 3 months because of the nature of their abstinence. The first group could have had sex in the study period, but happened to have no such activity. Thus, the number of observed zeros is inflated by the structural zeros representing the abstinent girls in the second group, which cannot be explained in the same manner as the sampling zeros from the sexually active group. The negative binomial model does not distinguish between the two types of zeros, but ZIP and ZINB do.

Within the context of our study, the subjects who were continually abstinent from a type of behavior such as the unprotected vaginal sex during a given time period would have structural zeros as their outcomes. These subjects formed the nonrisk subgroup for the behavioral outcome under consideration, while the remaining subjects with either sampling zeros or positive count outcomes constituted the at-risk subgroup. The logistic regression module of ZIP models the probability of structural zeros, allowing us to assess whether the intervention had promoted abstinence from the risky behavior under study. The Poisson module models the mean frequency of the count outcome for the at-risk subgroup, providing information on the effect of the intervention for reducing the frequency of the sexual behavior for these subjects. Thus, when applying ZIP and ZINB to assess the intervention effect for our study, we obtain two sets of estimates: one contains information about the effect of the intervention for promoting abstinence, while the other for reducing the frequency of the behavior for those who continued to be at risk.

### 3.2. Model Comparisons

 Although ZIP and ZINB address structural zeros, it is difficult to tell whether they are the appropriate choice for the data at hand, since such zeros are latent and not directly observed. Thus, it is important to apply goodness of fit statistics to help guide the selection of models appropriate and optimal for the data.

In general, nested models are compared using likelihood or score test, while nonnested models are evaluated using the Vuong test [8, 9]. For the models considered: Poisson, NB, ZIP, and ZINB, Poisson is nested with NB, as discussed earlier. Thus, it follows that ZIP is nested within ZINB.

However, there is not yet a consensus on whether Poisson (NB) is nested with ZIP (ZINB). The Poisson (NB) is a one-component model for a single population, while ZIP (ZINB) is a two-component mixture model for a population consisting of two subpopulations. Thus, the two classes of models cannot be used to describe the same study population. Because of this, the Poisson (NB) and ZIP (ZINB) models are regarded by many as not being nested. Therefore, the log-likelihood ratio test and score test cannot be applied to compare these models [10–14].

On the other hand, others argue that the Poisson and NB models are nested within the ZIP and ZINB models, respectively, from the perspective of model transformation, and propose to test the nested structure using the likelihood ratio and score test [15, 16]. The augument that the one component models Poisson and NB are nested within their two-component models, ZIP and ZINB respectively, may be viewed. For example, as we presented above, as the amount of structural zero, or the probability *ω*, approaches 0, ZIP reduces to Poisson. However, the nested structure may not be tested using the standard approach such as the Wald, likelihood, and score statistics by simply setting *α* to zero, since zero is a boundary point of the range of *α*. Thus, modified likelihood ratio and score tests must be used [15, 17, 18].

We have no preference for one perspective over the other. In this manuscript, we do not treat Poisson (NB) as nested within ZIP (ZINB) and use the Vuong test to compare them.

Vuong proposed a general approach to model selection whether the competing models are nested, overlapping, or nonnested, and whether the models are correctly specified [8]. Vuong's statistic is the average log-likelihood ratio suitably normalized so that it can be compared to a standard normal. The test statistic is defined by

(8) $$V=\frac{\sqrt{n}\bar{m}}{S_{m}},$$

where *m*_*i*_ = log⁡[*f*_1_(*y*_*i*_)/*f*_2_(*y*_*i*_)] and *f*_1_ and *f*_2_ are two competing probability models such as Poisson versus ZIP within our context, $\bar{m}=\left(1/n\right)\sum_{i=1}^{n}m_{i}$, and $S_{m}^{2}=\left(1/(n-1)\right)\sum_{i=1}^{n}{(m_{i}-\bar{m})}^{2}$. The statistic has an asymptotically standard normal distribution and the test is directional, with a large positive (negative) value favoring *f*_1_(*f*_2_), and a value close to zero indicating that neither model fits the data well [8, 9].

We compare and choose the best model among the Poisson, NB, ZIP, and ZINB through the following steps. If the ZINB model is rejected in favor of the NB model by the Vuong test, then the null of no structural zero is not rejected, implying a single study population. In this case, we estimate the heterogeneity parameter *α* in the NB model. If this parameter is significant, it suggests that this dispersion parameter accounts for unobservable heterogeneity responsible for overdispersion. On the other hand, if the Vuong test shows that the NB is rejected in favor of the ZINB model, we then test if the parameter *α* in the ZINB model is significant. If the estimate of *α* is again significant, it shows that we have both structural zeros and extra Poisson variation.

To compare the predictive performance of Poisson, NB, ZIP, and ZINB models, various indices such as likelihood ratio, Akaike's information criterion (AIC) [19], Bayesian information criterion (BIC) [20], and Lagrange multiplier (LM) statistic can be used. In addition, we can compare the abilities of predicting the number of zeros and observed versus predicted probabilities among the competing models. The difference between the predicted and actual counts forms the basis of the mean squared error (MSE) performance measure.

AIC is used for comparing nonnested models. This statistic takes into consideration model parsimony penalizing for the number of predictors in the model, AIC = −2log *L *+ number of parameters. The first term is essentially the deviance and the second a penalty for the number of parameters. The smaller the AIC value, the better the model fit. A popular alternative is BIC, defined by BIC = −2log *L* + Log (number of cases) × number of Parameters. However, as BIC imposes a harsher penalty for the estimation of each additional covariate, it often yields oversimplified models.

 Lagrange multiplier (LM) statistic is also often used to directly test overdispersion within our context [21]. For example, if we consider Poisson regression as a special case of NB under the restriction with the mean equal to the variance, Greene's Lagrange multiplier (LM) statistic is $\text{LM}={\left(e^{\prime }e-n\bar{Y}\;\right)}^{2}/2u\prime u$, where *u* = exp⁡⁡(*X* ^*T*^ *β*) and *e* = *Y* − *u*. Under the null of Poisson, LM follows the chi-squared distribution with one degree of freedom.

 To compare the fit of the various models when applied to the current HIV risk reduction intervention data, we fitted Poisson, NB, ZIP, and ZINB regression models for each of the four primary outcomes at 3, 6, and 12 months, (1) all vaginal sex episodes (regardless of partner type or condom use status), (2) unprotected vaginal sex with steady partners, (3) unprotected vaginal sex with other partners, and (4) any unprotected vaginal sex with steady or other partners. For space consideration, we focus on the 12-month outcomes, with a brief summary of the analysis results of 3- and 6-month outcomes.

For all analyses, we controlled for the demographic variables of age, race, ethnicity, poverty, Hispanic, multiple race, as well as controlling for the baseline status of the dependent measure. Logistic regression (with treatment condition as the response) was performed to determine if baseline characteristics of the subjects would predict group assignment using the backward elimination procedure. These six covariates were derived based on the logistic regression, along with HIV risk prevention literature and our experience with this particular study population.

We fitted the Poisson and NB regression models using the same six covariates and respective baseline measures of the dependent variable. For ZIP and ZINB models, we retained all covariates used in the Poisson and NB models in both parts of the model, that is, the logistic and Poisson (or NB) for ZIP (ZINB). All statistical analyses and plots were performed using SAS NLMIXED, GENMOD procedures, and some user-written SAS Macros [22].

## 4. Results

Table 2 presents the results from the four models for *all vaginal sex episodes* outcome at 12 months, while controlling for the baseline value of the outcome, and six other covariates: age at baseline, white race (0 = not, 1 = yes), multiple racial (0 = no, 1 = yes), other ethnicity (0 = no, 1 = yes), Hispanic (0 = no, 1 = yes), poverty (0 = no, 1 = yes), and treatment condition (0 = control, 1 = intervention). For the two-component ZIP (ZINB) model, the table includes results from both the logit and Poisson (NB) modules. We tested for excess zeros by comparing the Poisson and NB models to the ZIP and ZINB models, respectively, using the Vuong test. The test statistics, *V* = 7.64 for ZIP versus Poisson and *V* = 3.24 for ZINB versus NB, show that both ZIP and ZINB provide a better fit than their one-component counterparts. The Lagrange multiplier is also significant. Hence, there is evidence of overdispersion due to excess zeros. Further, estimates of the dispersion parameter *α* = 0.84 from ZINB and *α* = 1.39 from NB also indicate overdispersion due to data clustering.

 For the nested structure, both NB and ZINB had a much lower −2 log likelihood than that of the Poisson and NB (*P* values < 0.0001). Thus, likelihood ratio tests also favor ZIP over Poisson, and ZINB over NB models.

The AIC obtained from the data were in the following order (see Table 2):

(9) $$\text{AI}{\text{C}}_{\text{ZINB}}<AI{\text{C}}_{\text{NB}}\ll \text{AI}{\text{C}}_{\text{ZIP}}\ll \text{AI}{\text{C}}_{\text{Poisson}}.$$

The BICs showed the same order. Thus, under both AIC and BIC, ZINB seems to be optimal model among the four models considered.

 For the results at 3 and 6 months, the Vuong and likelihood ratio tests, and AIC criterion, also show that ZINB (NB) was a better fit of the data than ZIP Poisson, with ZINB having the lowest AIC among the four models. In addition, both dispersion parameter and Lagrange multiplier (LM) tests implied the existence of overdispersion due to data clustering. The results from BIC are consistent with those from AICs, with the exception that NB at 6 month had a slightly smaller BIC than that of ZINB (3751.12 versus 3757.00).

 Next we compared the four models in terms of how well each model captures the zeros in the data.  Table 3 summarizes the percentage of zeros captured by the Poisson, NB, ZIP, and ZINB. For the *all vaginal sex episodes* outcome at 3 and 12 months, the fitted zeros by ZIP were very close to the observed ones; at 6 months, ZINB was slightly better than ZIP in that regard. For all the visits, Poisson was the worst in estimating the zeros. For the *unprotected vaginal sex with steady partners*, the percentage of zeros estimated by ZIP had almost an exact match to their observed counterparts at 3, 6, and 12 months. Compared to ZIP, the estimated percents of zeros by NB and ZINB were slightly lower than that by ZIP. For the *unprotected vaginal sex with other partners *outcome, NB, ZIP, and ZINB all had good performance in estimating the zeros, with ZINB (ZIP) providing the best estimate at 3 (6 and 12) months. For the *any unprotected vaginal sex with steady or other* partners outcome, ZIP performed the best, while ZINB slightly overestimated zeros at 12 months. Again, Poisson performed the worst.

**Figure 2:**
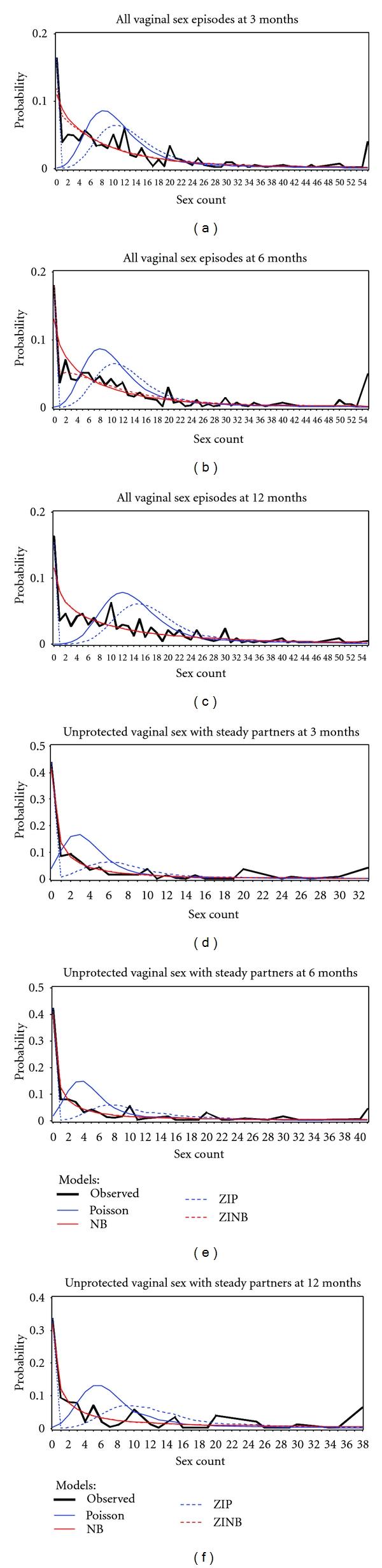
Comparisons between observed and predicted probabilities from four models for all types of vaginal sex episodes and unprotected vaginal sex with steady partners.

Plots of observed versus fitted values are also quite helpful to visualize model fit. For the count data within our context, we can compare the fitted and observed probabilities of the count response by taking the probability distribution into consideration. Shown in Figure 2 are the plots of the probabilities from the fitted models versus the observed for the *all vaginal sex episodes* and the *unprotected vaginal sex with steady partners* outcomes at 3, 6, and 12 months. ZINB fit the observed data well for all 3, 6, and 12 months, as compared to the other models. In terms of capturing the observed zeros, ZIP behaved very well overall across all three visits, while ZINB had the best fit to the zeros at 6 months.

Generally, the two-component nature of ZIP and ZINB provides them a competitive edge in terms of accurately representing the zeros in the data. Poisson exhibited the worst fit to both zero and positive counts, followed by ZIP. For example, for the all *vaginal sex episodes outcome* at 3-month visit, Poisson underestimated zeros and small counts (e.g., 0 ≤ count ≤ 4), but overestimated intermediate counts (e.g., 6 ≤ count ≤ 12); ZIP also underestimated small counts (e.g., 1 ≤ count ≤ 6) and overestimated intermediate counts (e.g., 7 ≤ count ≤ 12), although it fared better in the overestimated intermediate counts compared to the Poisson. NB underestimated zeros and overestimated small counts (e.g., 1 ≤ count ≤ 5 in same case), although with less bias than the Poisson.

ZINB was better than NB in both estimating the zeros and small counts, but it still underfitted the number of zeros, and overfitted the small counts (e.g., 1 ≤ count ≤ 5) at 3 month visit, but the fit improved at 6 month visit. At 12 months, ZINB and NB were identical, with both underestimating the number of zeros and overestimating the small counts (e.g., 1 ≤ count ≤ 5); the Poisson severely underestimated both zeros and small counts (1 ≤ count ≤ 5) but overestimated for intermediate counts (7 ≤ count ≤ 22). 

The performance improved for all these four models as the number of zeros decreased and the range of counts became smaller. For other outcomes, the plots for comparing the fitted and observed data and conclusions about the comparisons are quite similar and thus are not further discussed.

Taken together, ZINB is the best model in terms of model fit by best capturing the shape of distribution of observed values at the same time, followed by NB, ZIP, and the Poisson. The results indicate that there are not only structure zeros presented in the data, but data clustering as well. This conclusion is consistent with the goal of the intervention and objects of this study—to promote safer sex and abstinence from risky sexual behaviors. Thus, the better performance of the two-component ZIP and ZINB models over their respective one-component counterpart Poisson and NB is expected from the conceptual grounds.

Upon establishing the right models, we now turn our attention to the interpretation of the results with the specific context of the HIV prevention intervention study. As only ZIP and ZINB are appropriate for modeling the outcomes in this study, they were fit to the data at 3, 6, and 12 months data for each outcome. For illustration purposes and space consideration, we focus on the intervention results for the *all types of vaginal sex episodes* outcome at 12 months.

Both models were fit, while controlling for the baseline value of the outcome, and the six covariates. We did not model all followup data simultaneously using longitudinal methods, since such an approach was unavailable from major software packages such as SAS, which we used to fit ZIP and ZINB in the current context. Rather, we modeled each followup visit one at a time, while controlling for the outcome of interest at baseline along with the covariates mentioned above. Also, we only report the results for the treatment condition as the intervention effect is the main outcome of this randomized controlled trial.

Table 4 displays the estimates of regression coefficients for the intervention effect of both components of the ZIP and ZINB models, respectively. Shown under the *Poisson regression part from ZIP (negative binomial regression part from ZINB)* are the coefficients for the treatment condition, with the control condition serving as a referent level, for the Poisson submodel of ZIP (Negative binomial submodel of ZINB) over each of the followup visits for the *All types of vaginal sex episodes* outcome. Shown under the *Logistic regression part* are the coefficients for the logistic regression submodel of ZIP (ZINB). As mentioned, the Poisson (Negative binomial) component of ZIP (ZINB) models the effect of the intervention for the at-risk subgroup, while the logistic module models the intervention effect for the nonrisk subgroup.

 Using ZIP, the effect of the intervention condition was statistically significant for the Poisson module over all the followup visits (*P* values < 0.0001). The negative sign of the coefficient indicates that the intervention reduced the mean frequency of this outcome for the subjects in the at-risk group who received the intervention, as compared to those within the control group. The reduction was 13.89% (1−exp (−0.1495) = 0.1389), 12.83%, and 13.06%, at 3, 6, and 12 months, respectively. The effect of the intervention condition was also observed for the negative binomial component of ZINB over all the followup visits, although the results were only significant at 3 and 12 months with *P* values = 0.0055, and 0.0486, respectively. As compared with the control condition, the reduction was 19.87% (1−exp (−0.2215) = 0.1987), 13.13%, and 17.40%, at 3, 6, and 12 months, respectively.

The intervention effect was also statistically significant for the logistic model from ZIP and ZINB at 6 months with *P* value = 0.0011, and 0.0044, respectively. The positive sign of the coefficient indicates that a significantly higher proportion of girls stayed abstinent from the particular type of sex under consideration in the intervention than in the control group, with an odds ratio of 2.19 (log odds ratio = 0.7859) from ZIP model, and 2.74 (log odds ratio = 1.0068) from ZINB. Although the intervention effect did not reach statistical significance at 3 and 12 months, the positive signs of the coefficient at both visits from ZIP, and 12 month visit from ZINB, show that more girls in the intervention group exercised abstinence than those in the control group during the respective time periods.

## 5. Discussion

In this paper, we have compared four regression models for count data: Poisson, NB, ZIP, and ZINB. We demonstrated the superior performances of ZINB and ZIP, when applied to data from a randomized controlled HIV risk reduction intervention study for a high-risk population of urban adolescent girls. We have found from the analyses that ZINB provides a better fit than Poisson, NB, and ZIP, under Vuong's test, likelihood ratio test, AIC and BIC criteria.

Our data have two features that are common in studies on this research topic: a preponderance of zeros and overdispersion. The Poisson, due to its restrictive assumption (variance equals to its mean), is not suitable for modeling this kind of data. Although NB addresses overdispersion by including a dedicated dispersion parameter, the inclusion of this parameter seems to artificially cause an increase of the probabilities of both zero and positive counts, without improving the fit for modeling the data. An interesting phenomenon we observed in this regard is that NB is capable of predicting large percents of zeros when the count range is not too large, even better than ZINB. For example, for the *unprotected vaginal sex with other partners* outcome where the count ranged from 0 to 9, the AIC of NB was only slightly higher than that of ZINB.

The difference between NB and ZINB appears to be due to the way in which these two types of models accommodate variability caused by a preponderance of zero-valued observations, and whether the model assumed for the mean count response is reasonable. In our example, NB underestimates the amount of zeros as well small positive counts, because it addresses the presence of structural zeros by increasing its variance through the dispersion parameter [21]. ZIP is more capable of modeling extra zeros than either Poisson or NB. The limitation of ZIP lies in its Poisson component, which cannot address overdispersion due to data clustering. In our case, ZIP underpredicted small, but overpredicted moderate counts for the *All types of vaginal sex episodes *outcome. Even if the data range was not large, ZIP still underpredicted small counts and overpredicted the moderate counts, as compared to other models in the case of the *unprotected vaginal sex with other partners* outcome (not shown).

Since the purpose of the HIV risk reduction intervention study is to test the hypothesis that adolescent girls receiving the HIV risk reduction intervention would reduce their risky sexual behaviors as well as increase the rate of abstinence of such behaviors as compared to those in the control condition, ZIP seems an appropriate approach on this conceptual ground. However, we must keep in mind that ZIP does not address overdispersion due to data clustering. This limitation is demonstrated by the smaller standard errors and thus smaller *P*-values in our reported ZIP results. ZINB on the other hand addresses both issues due to its dual capability of modeling structural zeros and overdispersion at the same time.

 In spite of its superior performance in fitting these data, ZINB is not without limitations. For example, ZINB not only underpredicted the zeros but also in some cases overpredicted the zeros, such as in our analysis of the *unprotected vaginal sex with other partners* outcome, in which case ZINB underpredicted zeros at 3 month, but over predicted zeros at 12 months (not shown). These limitations likely stem from their assumptions of distributions, and as such distributions-free ZIP and ZINB forgoing the Poisson and NB assumptions will address the limitations.

Another major limitation is the cross-sectional analysis performed for each followup visit, despite the longitudinal study data, due primarily to the unavailability of appropriate software for the latter data type from popular packages such as SAS and R. This limited our ability to formally test the findings that the percents of reported sex activity decreased from 3 to 12 months for all these four outcomes. Our future work will focus on extending our comparisons of different models and even developing distribution-free alternatives to a longitudinal data setting.

## Figures and Tables

**Table 1:** Distribution of outcomes.

Outcome	Zero (percentage)	Mean	Variance
All vaginal sex episodes			
3 months	16.35	12.00	183.53
6 months	17.90	12.01	214.77
12 months	16.28	15.26	326.26
Unprotected vaginal sex with steady partners			
3 months	43.67	4.90	70.01
6 months	42.38	6.00	108.70
12 months	33.69	7.15	112.04
Unprotected vaginal sex with other partners			
3 month	87.69	0.32	1.31
6 months	89.61	0.27	1.15
12 months	86.50	0.39	1.69
Any unprotected vaginal sex with steady or other partners			
3 months	39.47	4.80	52.65
6 months	39.67	5.60	72.99
12 months	29.44	7.55	122.98

**Table 2:** The estimated parameters, coefficients of the Poisson, NB, ZIP, and ZINB models for all *vaginal sex episodes* outcome at 12 months.

Parameter	Poisson	Negative binomial	Zero-inflated poisson		Zero-inflated negative binomial	
			Poisson	Logit	NB	Logit
Intercept	1.5424*	1.7947*	2.0453	−0.8089	2.0630*	−0.1824
Age	0.0491*	0.0290	0.0392*	−0.0476	0.0252	−0.0781
white	0.1406*	0.1914	0.1077*	−0.0588	0.1863	0.0773
Multiracial	0.4249*	0.3576**	0.3165*	−0.8917	0.2679	−1.0362
Other race	0.0275	−0.0222	0.2054	0.1203	0.0388	0.3403
Hispanic	−0.2908*	−0.1882	−0.1976*	0.5028	−0.1406	0.4350
Poverty	0.2060*	0.2899*	0.1600*	−0.3786	0.2232*	−0.4106
Baseline	0.0163*	0.0181*	0.0147*	−0.0206∗	0.0159*	−0.0227
Condition	−0.1542*	−0.2062**	−0.1400*	0.1227	−0.1911*	0.0813
Dispersion		1.3873*			0.8370*	
Lagrange multiplier	63120.17*					
−2 Log Likelihood	9244.29	3512.26	7344.30		3474.70	
Parameter	9	10	18		19	
AIC	9262.29	3532.26	7380.00		3512.70	
BIC	9299.83	3573.98	7455.10		3591.90	
Vuong test			7.6369*		3.2400*	

*Indicates that the coefficients are significant at 5%; **the coefficients are significant at 10%.

**Table 3:** Percentage of zeros captured by the POIS, NB, ZIP, and ZINB models.

Outcome	Observed	POIS	NB	ZIP	ZINB
All types of vaginal sex episodes					
3 months	16.353	0.0437	10.968	16.353	11.932
6 months	17.897	0.0417	13.134	16.974	17.877
12 months	16.284	0.0021	11.578	15.449	11.578
Unprotected vaginal sex with steady partners					
3 months	43.667	3.836	41.545	43.663	41.545
6 months	42.379	1.664	40.358	42.379	40.358
12 months	33.689	0.298	32.337	33.688	32.337
Unprotected vaginal sex with other partners					
3 month	87.608	73.448	87.608	87.516	87.743
6 months	89.610	77.570	89.533	89.565	89.468
12 months	86.498	69.631	86.344	86.481	86.311
Any unprotected vaginal sex with steady or other partners					
3 months	39.474	3.322	36.825	39.469	38.617
6 months	39.668	1.753	37.016	39.666	37.016
12 months	29.436	0.225	28.576	29.436	29.597

**Table 4:** Intervention effect for all types of vaginal sex episodes outcome from ZIP and ZINB.

Variables	Coefficient of intervention with control as a reference	Standard Error	Chi-square	*P* value
Poisson regression part from ZIP				
3 months	−0.1495	0.0255	34.44	<0.0001
6 months	−0.1373	0.0252	29.68	<0.0001
12 months	−0.1400	0.0238	34.65	<0.0001
Negative binomial part from ZINB				
3 month	−0.2215	0.0795	−2.79	0.0055
6 months	−0.1407	0.0888	−1.58	0.1138
12 months	−0.1911	0.0966	−1.98	0.0486
Logistic regression Part from ZIP				
3 months	0.2252	0.2409	0.87	0.3499
6 months	0.7859	0.2407	10.66	0.0011
12 months	0.1227	0.2534	0.23	0.6282
Logistic regression part from ZINB				
3 months	−0.2144	0.5684	−1.22	0.2208
6 months	1.0068	0.3521	2.86	0.0044
12 months	0.0813	0.3306	0.25	0.8059
